# Fluconazole Monotherapy Is a Suboptimal Option for Initial Treatment of Cryptococcal Meningitis Because of Emergence of Resistance

**DOI:** 10.1128/mBio.02575-19

**Published:** 2019-12-03

**Authors:** William Hope, Neil R. H. Stone, Adam Johnson, Laura McEntee, Nicola Farrington, Anahi Santoro-Castelazo, Xuan Liu, Anita Lucaci, Margaret Hughes, Jason D. Oliver, Charles Giamberardino, Sayoki Mfinanga, Thomas S. Harrison, John R. Perfect, Tihana Bicanic

**Affiliations:** a Antimicrobial Pharmacodynamics and Therapeutics, University of Liverpool, Liverpool Health Partners, Liverpool, United Kingdom; b Royal Liverpool Broadgreen University Hospital Trust, Liverpool Health Partners, Liverpool, United Kingdom; c Institute of Infection and Immunity, St. George’s, University of London, London, United Kingdom; d Centre for Genomics Research, University of Liverpool, Liverpool, United Kingdom; e F2G Ltd., Eccles, United Kingdom; f Division of Infectious Diseases and International Health, Duke University School of Medicine, Durham, North Carolina, USA; g National Institute of Medical Research, Dar es Salaam, Tanzania; Centers for Disease Control and Prevention

**Keywords:** antifungal resistance, antimicrobial resistance, cryptococcus, fluconazole, fungus, meningitis, yeast

## Abstract

Cryptococcal meningitis is a lethal disease with few therapeutic options. Induction therapy with fluconazole has been consistently demonstrated to be associated with suboptimal microbiological and clinical outcomes. Exposure to fluconazole causes dynamic changes in antifungal susceptibility, which are associated with the development of aneuploidy. The implications of this phenomenon for pharmacodynamics of fluconazole for cryptococcal meningitis are poorly understood. The pharmacodynamics of fluconazole were studied using a hollow-fiber infection model (HFIM) and a well-characterized murine model of cryptococcal meningoencephalitis. The relationship between drug exposure and both antifungal killing and the emergence of resistance was quantified. The same relationships were further evaluated in a recently described group of patients with cryptococcal meningitis undergoing induction therapy with fluconazole at 800 to 1,200 mg/day. The pattern of emergence of fluconazole resistance followed an “inverted U.” Resistance amplification was maximal and suppressed at ratios of the area under the concentration-time curve for the free, unbound fraction of the drug to the MIC (*f*AUC:MIC) of 34.5 to 138 and 305.6, respectively. Emergence of resistance was observed *in vivo* with an *f*AUC:MIC of 231.4. Aneuploidy with duplication of chromosome 1 was demonstrated to be the underlying mechanism in both experimental models. The pharmacokinetic (PK)-pharmacodynamic model accurately described the PK, antifungal killing, and emergence of resistance. Monte Carlo simulations from the clinical pharmacokinetic-pharmacodynamic model showed that only 12.8% of simulated patients receiving fluconazole at 1,200 mg/day achieved sterilization of the cerebrospinal fluid (CSF) after 2 weeks and that 83.4% had a persistent subpopulation that was resistant to fluconazole. Fluconazole is primarily ineffective due to the emergence of resistance. Treatment with 1,200 mg/day leads to the killing of a susceptible subpopulation but is compromised by the emergence of resistance.

## Abstract (precis)

Cryptococcal meningitis is a lethal disease with few treatment options. The incidence remains high and intricately linked with the HIV/AIDS epidemic. In many parts of the world, fluconazole is the only agent that is available for the initial treatment of cryptococcal meningitis despite considerable evidence that it is associated with suboptimal microbiological and clinical outcomes. Fluconazole has a fungistatic mode of action: it predominantly inhibits growth rather than causing fungal killing. Our work shows that the pattern of fluconazole activity is caused by the emergence of resistance in *Cryptococcus* not detected by standard susceptibility tests, with chromosomal duplication/aneuploidy as the main mechanism. Resistance emergence is related to drug exposure and occurs with the use of clinically relevant regimens. Hence, fluconazole (and potentially other agents that target 14-alpha-demethylase) is compromised by an intrinsic property that limits its effectiveness. However, this resistance may be potentially overcome by dosage escalation or the use of combination therapy.

## INTRODUCTION

Fluconazole is a mainstay for the treatment of cryptococcal meningitis: it has an extensive safety usage database, is orally bioavailable, has excellent central nervous system (CNS) penetration, has few clinically relevant drug-drug interactions, and has a plethora of clinical data for its use. Unlike amphotericin B and flucytosine (5FC), the other two antifungal agents recommended for therapy of cryptococcal meningitis (1), fluconazole is the only drug available in much of Africa, where cryptococcal meningitis is most prevalent (2). However, fluconazole monotherapy, even when used at high doses of 800 to 1,200 mg/day (3), is associated with low rates of fungal clearance in the cerebrospinal fluid (CSF) and suboptimal clinical outcomes compared with amphotericin B-based therapy or combinations with flucytosine. One-year mortality approaches 70% in African cohorts (4–7). Historically, this has been attributed to a fungistatic pattern of antifungal activity, whereby inhibition of growth is observed rather than orders of logarithmic CSF yeast killing. Recent insights into the pharmacodynamics of fluconazole against Cryptococcus neoformans (8) and a better understanding of the molecular mechanisms of resistance (9) suggest that this concept is overly simplistic.

Primary cryptococcal resistance to high-level fluconazole caused by a stable heritable genetic mechanism appears to be relatively rare. However, clinical relapses with isolates possessing reduced susceptibility to fluconazole commonly occur in the setting of monotherapy (10). For instance, C. neoformans displays heteroresistance to fluconazole; i.e., there is a resistant subpopulation present even in the absence of the drug, which is so small (often <1%) that it may be missed by conventional antifungal susceptibility (MIC) testing. Following drug exposure, this subpopulation expands (or at least persists) during drug therapy, while the susceptible yeast subpopulation is killed (11). Partial and complete duplications of chromosome 1 are most commonly observed in this resistant subpopulation; however, other disomies have also been reported (12, 13). While aneuploidy has been demonstrated in preclinical (12) and, more recently, clinical (13) contexts, the pharmacodynamics (PD) of this phenomenon are not well understood. A better understanding of the relationship between fluconazole exposure and the emergence of resistance is required to further optimize fluconazole-based regimens for the treatment of cryptococcal meningitis.

Several studies have examined the relationship between *in vitro* fluconazole susceptibility and clinical outcomes for informing clinical breakpoints (14). Others have formally described the population pharmacokinetics (PK) of fluconazole in human plasma and CSF (15). However, none have combined fungal *in vitro* susceptibility testing and molecular mechanisms of resistance with direct antifungal drug exposures at the site of infection and then linked these variables with microbiological outcomes such as fungal killing and the emergence of resistance. In this study, we used several preclinical experimental platforms and a clinical PK-PD study to explore the pharmacodynamics of fluconazole in the treatment of cryptococcal meningitis when administered as induction monotherapy. We used a recently described hollow-fiber infection model (HFIM) of cryptococcal meningitis (16) in which simulation of concentration-time profiles of fluconazole in CSF enables the full exposure-response relationships for fluconazole-susceptible and -resistant subpopulations at the site of infection to be delineated. These *in vitro* findings were then confirmed in a well-characterized murine model of cryptococcal meningitis and further linked to a clinical PK-PD substudy.

## RESULTS

### Strains, mutational frequency of resistance, and detection of resistance.

**FIG 1:**
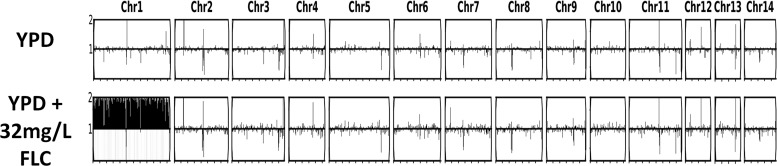
Association between aneuploidy and the ability of C. neoformans to grow on agar containing 32 mg/liter fluconazole (FLC). Sequence reads are mapped against the reference genome. Blackout of a chromosome indicates a 2-fold increase in copy number.

H99 (ATCC 208821) was used as the challenge strain in the hollow-fiber infection model and murine model of cryptococcal meningitis. The fluconazole MIC was 4 mg/liter using Clinical and Laboratory Standards Institute (CLSI) microdilution methodology (17). The mutational frequency of resistance was 8 × 10^−4^ at 32 mg/liter of fluconazole. Concentrations of 32 and 64 mg/liter were incorporated into yeast extract-peptone-dextrose (YPD) agar for the detection of resistance in the hollow-fiber and murine experiments. Aneuploidy of chromosome 1 was observed in isolates able to grow on agar containing fluconazole at 32 mg/liter (Fig. 1).

### Hollow-fiber infection model.

**FIG 2:**
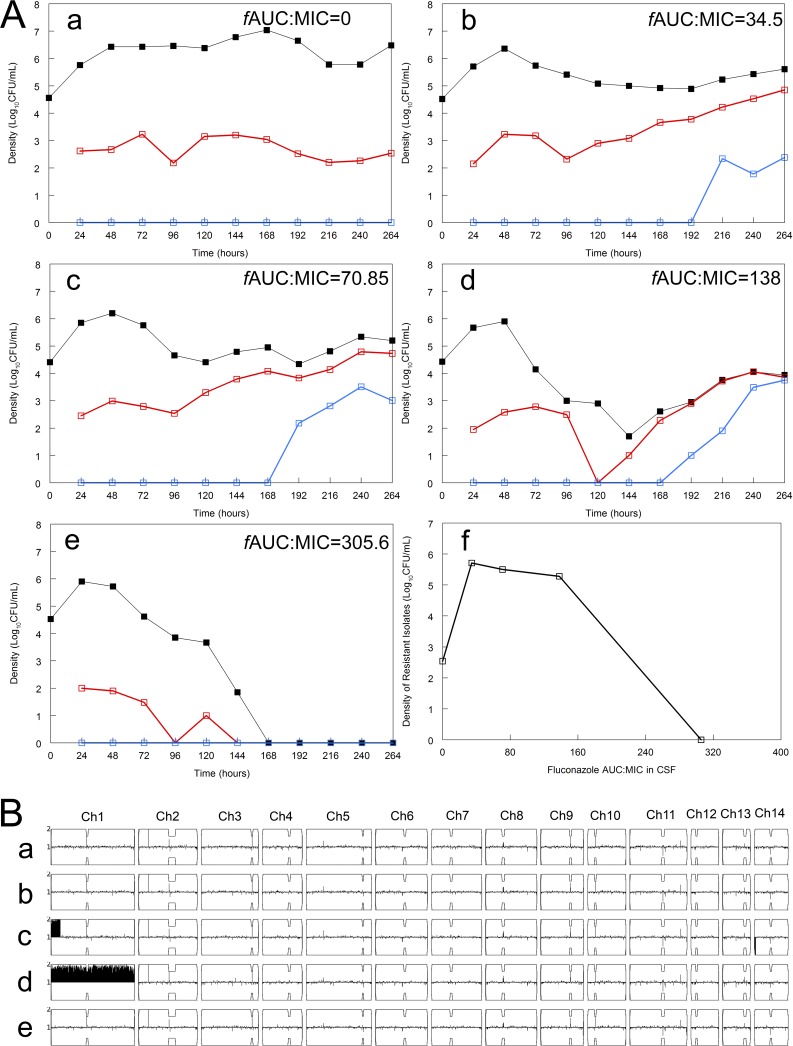
(A) Pharmacodynamics of fluconazole against Cryptococcus neoformans H99 (fluconazole MIC of 4 mg/liter) in a hollow-fiber infection model. Treatment was delayed for 24 h postinoculation. (a to e) Time courses of different populations in response to different concentration-time profiles of fluconazole in CSF that mimic those in humans. Black, total cryptococcal population; red, resistant subpopulation able to grow on agar containing fluconazole at 32 mg/liter; blue, resistant subpopulation able to grow on agar containing fluconazole 64 mg/liter. (f) The relationship between *f*AUC:MIC and the resistant fungal density at the end of the experiment shows an “inverted U.” (B) Chromosome map (Y-map). (a) Ploidy map of the inoculating strain (before fluconazole exposure) showing monosomy. Colonies taken from drug-free YPD agar do not show aneuploidy, but a colony taken from fluconazole-containing agar shows disomy of chromosome 1. (b to e) Y-maps of colonies taken at the end of the hollow-fiber infection model experiment (264 h for all arms except arm e, for which 144 h was the last culture-positive time point. In arm c, partial duplication of chromosome 1 is apparent, while complete disomy of chromosome 1 is seen in arm d. The duplicated region of chromosome 1 in arm c contains the *ERG11* gene, the fluconazole target. For the control (arm a), the lowest dose (arm b), and the highest dose (arm e), no aneuploidy is observed in unselected colonies, suggesting that amplification of the resistant, chromosome 1 disomic subpopulation occurs with moderate exposures to fluconazole.

The shape of the fluconazole concentration-time profile in human CSF was reproduced in the hollow-fiber infection model. Different concentration-time profiles with the same overall shape were generated to enable the pharmacodynamics for both susceptible and resistant subpopulations to be explored and quantified. Treatment was delayed for 24 h postinoculation. Low exposures of fluconazole resulted in some decrease in the total fungal burden, but this was offset by the emergence of a resistant subpopulation that was able to grow on agar containing fluconazole at 32 and 64 mg/liter (Fig. 2A, panel b). For a ratio of the area under the concentration-time curve for the free, unbound fraction of the drug to the MIC (*f*AUC:MIC) in the range 34.5 to 138 (Fig. 2A, panels c and d), almost all of the fungal burden at the end of the experiment consisted of resistant yeasts able to grow on agar containing fluconazole at 64 mg/liter. In the case of Fig. 2A, panel d, 93% of the total population was highly resistant. Fluconazole exposures of an *f*AUC:MIC of ≥305.6 resulted in the killing of both the susceptible and resistant subpopulations and apparent sterilization of the cartridge (Fig. 2A, panel e). A plot of the *f*AUC:MIC within the CSF versus the fungal burden of the resistant colonies at the end of the experiment revealed an “inverted U,” which is characteristic of many drug exposure-versus-resistance relationships (18) (Fig. 2A, panel f).

Samples from the cartridge at the beginning and end of the HFIM experiment were cultured on drug-free YPD (i.e., unselected) agar incubated at 30°C in air. A sweep of colonies was selected for sequencing. Unselected isolates at the beginning of the experiment (prior to exposure to fluconazole) did not have aneuploidy (Fig. 2B, panel a). Similarly, colonies from the control arm at the end of the experiment did not have aneuploidy (Fig. 2B, panel b). In contrast, unselected colonies obtained at the end of the experiment from arms c and d (*f*AUC:MIC of 70.85 and 138, respectively) had aneuploidy with partial and complete duplications of chromosome 1, respectively. Both duplications involved the *ERG11* gene, which encodes the triazole target lanosterol 14-α-demethylase. Unexpectedly, aneuploidy was not demonstrated in arm b despite the emergence of phenotypic resistance, which may have been due to either missing the resistant subpopulation (i.e., a smaller fraction of resistant organisms than in arms c and d) or an alternative molecular mechanism of resistance unrelated to aneuploidy.

### Murine studies of cryptococcal meningoencephalitis.

The *in vivo* pharmacodynamics of fluconazole against Cryptococcus neoformans were studied in a well-characterized murine model of meningoencephalitis. PK-PD relationships were established over the course of multiple independently conducted experiments. The dosages of fluconazole used for the PK and PD studies were determined from preliminary experiments (see Fig. S1 in the supplemental material) and previously reported studies (8).

10.1128/mBio.02575-19.1 FIG S1 Time course of fungal burden in the brain of mice inoculated with Cryptococcus neoformans H99 and receiving fluconazole. The drug was administered daily via gavage. Treatment commenced at 24 h postinoculation. Data are means ± standard deviations for 3 mice. Download FIG S1, TIF file, 0.6 MB. Copyright © 2019 Hope et al. 2019 Hope et al. This content is distributed under the terms of the Creative Commons Attribution 4.0 International license.

**FIG 3:**
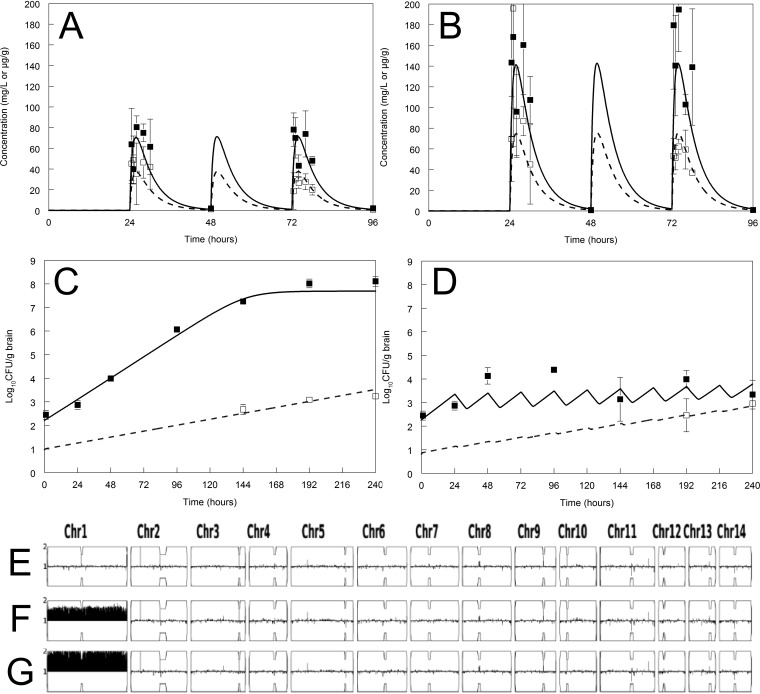
Representative murine PK-PD experiments. (A and B) Murine PK in plasma (raw data are shown as solid squares, and model fit is shown with a solid line) and cerebrum (open squares and broken line) following the administration of fluconazole at 125 and 250 mg/kg/day, respectively, by oral gavage. Data are means and standard deviations for 3 mice. (C and D) Pharmacodynamics for controls (C) and fluconazole at 250 mg/kg/day (D). The closed and open squares are the raw data from the total and drug-resistant subpopulations, respectively. For both controls (C) and drug-treated mice (D), emergence of resistance was not observed until relatively late in the experiment. The solid and broken lines are the model fits from the mean Bayesian posterior estimates for the total and resistant subpopulations, respectively. The bottom panel shows a Y-map (chromosome map) of Cryptococcus neoformans H99 grown from brain homogenates. (E) There was no resistant subpopulation within the inoculum because it was either truly absent or below the limit of detection. (F) Colonies from control mice growing on fluconazole-containing agar had aneuploidy at 192 h. (G) Colonies from mice receiving fluconazole at 250 mg/kg/day and growing on fluconazole-containing agar had aneuploidy at 192 h. Sequencing of colonies growing on fluconazole-containing agar showed disomy of the chromosome in all cases.

The pharmacokinetics of fluconazole in plasma and cerebrum using dosages of 125 and 250 mg/kg of body weight/day administered by oral gavage are shown in Fig. 3A and B. A dose of 250 mg/kg/day was limited by the maximum volume that can be administered by oral gavage under the terms of our laboratory animal license. The total plasma drug AUC:MIC value associated with the administration of 250 mg/kg/day was 260 (i.e., an *f*AUC:MIC of 231.4 assuming 11% protein binding in the mouse). The dose-response relationship using the total fungal burden as the pharmacodynamic endpoint was established using 25, 50, 125, and 250 mg/kg/day orally (Fig. S1). The emergence of resistance was explored using a destructive design with controls and a single dosage of fluconazole at 250 mg/kg day orally and plating murine brain homogenates onto YPD agar containing fluconazole at 32 mg/liter (Fig. 3C and D). A dosage of 250 mg/kg/day consistently resulted in a logarithmic reduction of the cerebral fungal burden relative to controls (Fig. 3D). The emergence of resistance was observed in untreated controls in a fixed proportion to the total fungal burden (Fig. 3C). The emergence of resistance (defined as the ability to grow on agar containing fluconazole at 32 mg/liter) was observed in mice receiving 250 mg/kg/day in the second week of the experiment, at between 192 and 240 h postinoculation, which was consistent with observations from the HFIM. The use of agar containing fluconazole at 64 mg/liter resulted in inconsistent results, where some experiments showed an emergence of resistance, while others did not (Fig. S2). The variability in the emergence of resistance in these experiments is probably explained by cells with aneuploidy not being within the CNS immediately postinoculation, or the *in vivo* rate of development of duplication not present immediately postinoculation was >240 h. A higher fungal density (i.e., greater than the mutational frequency) at the time of treatment initiation may have resulted in more consistency in the emergence of resistance. This could have been facilitated by delaying the initiation of therapy or inoculation directly into the CNS as described by others (see, for example, reference 19). The chromosomal ploidy map from colonies sequenced from murine brain homogenates plated onto fluconazole-containing agar demonstrated the emergence and persistence of chromosome 1 disomy after >96 h of fluconazole exposure (Fig. 3B).

10.1128/mBio.02575-19.2 FIG S2 Replicate experiments examining the pharmacodynamics of emergence of resistance. Here, resistance was defined as the ability to grow on agar containing fluconazole at 64 mg/liter. The same regimen of fluconazole was used. In panel A, there is emergence of resistance, whereas in panel B, there was none. This may be caused by a resistant subpopulation present at inoculation in panel A but not in panel B. Conversely, the emergence of a resistant clone upon therapy is a stochastic event that occurs in some but not all experiments. Download FIG S2, PDF file, 2.5 MB. Copyright © 2019 Hope et al. 2019 Hope et al. This content is distributed under the terms of the Creative Commons Attribution 4.0 International license.

The parameters for the mathematical model that links the dosage of fluconazole with plasma concentrations, concentrations in brain, the decline in total fungal burden in the brain, and the emergence of a resistant subpopulation are summarized in Table 1. The Bayesian posterior values from each individual (i.e., cohort of mice under the same experimental conditions) were used to fit the mathematical model to the PK and PD data shown in Fig. 3.

**TABLE 1:** Parameter values and dispersions from the mathematical model fitted to murine PK-PD data

Parameter^*a*^	Value		
	Mean	Median	SD
*K_a_* (h^−1^)	0.974	0.974	0.052
SCL/*F* (liters/h)	0.006	0.006	0.000
*V*/*F* (liters)	0.008	0.008	0.001
Kcp (h^−1^)	2.391	2.391	0.026
Kpc (h^−1^)	15.667	15.667	4.741
Kcs (h^−1^)	18.853	18.853	5.959
Ksc (h^−1^)	11.872	11.872	1.736
*V*cns/*f* (liters)	0.024	0.024	0.009
Kgmax_s (log_10_ CFU/g/h)	0.078	0.080	0.014
popmax (CFU/g)	26,294,350.171	42,296,494.976	22,574,600.414
kkmax_s (log_10_ CFU/g/h)	0.257	0.279	0.022
C_50_k_s (mg/liter)	25.090	23.496	1.641
Hk_s	15.252	14.323	1.167
Kgmax_r (log_10_ CFU/g/h)	0.032	0.026	0.007
kkmax_r (log_10_ CFU/g/h)	1.469	1.716	0.436
C_50_k_r (mg/liter)	88.795	89.265	1.809
Hk_r	18.989	19.822	0.950
IC_s (CFU/g)	148.570	144.106	17.730
IC_r (CFU/g)	9.230	10.000	1.493

a Parameters are as follows: *K_a_* is the first-order rate constant that connects the gut to the bloodstream; SCL is the first-order clearance of fluconazole from the central compartment (i.e., bloodstream); *V* is the volume of the central compartment; Kcp and Kpc are the first-order intercompartmental rate constants that connect the central and peripheral compartments; Ksc and Kcs are the first-order intercompartmental rate constants that connect the central compartment and CSF; *V*cns is the volume of the CSF; *F* is the oral bioavailability of fluconazole; Kgmax_s and Kgmax_r are the maximum growth rates of the susceptible and resistant subpopulations, respectively; popmax is the maximum theoretical fungal density in the cerebrum; kkmax_s is the maximum rate of fluconazole killing of the susceptible subpopulation in the cerebrum; C_50_k_s is the cerebral concentration of fluconazole at which the rate of killing of the susceptible subpopulation is half maximal; Hk_s is the slope function for the effect of fluconazole on the susceptible population; kkmax_r is the maximum rate of fluconazole killing of the resistant subpopulation in the cerebrum; C_50_k_r is the cerebral concentration of fluconazole at which the rate of killing of the resistant subpopulation is half maximal; Hk_r is the slope function for the effect of fluconazole on the resistant population; and IC_s and IC_r are the estimated initial densities of the susceptible and resistant subpopulations in the cerebrum, respectively.

### Clinical study of fluconazole as induction therapy for cryptococcal meningoencephalitis.

A total of 17 patients receiving fluconazole as induction therapy for cryptococcal meningitis were enrolled and studied in Tanzania. The clinical characteristics are shown in Table 2. Patients received 800 to 1,200 mg fluconazole orally (or intravenously [i.v.]) as induction therapy in a single or divided dosage administered every 12 h.

**TABLE 2:** Characteristics of the 16 patients included in the pharmacokinetic study

Parameter	Value
Total no. of patients included	16
No. of male patients	6
No. of female patients	10
Age range (yr)	19–56
Median no. of CD4 cells/mm^3^ (IQR^*a*^)	28 (142–83)
No. (%) of patients with GCS^*b*^ < 15	8 (50)
No. (%) of patients with CSF opening pressure of >20 cm H_2_O	10 (63)
Median baseline fungal burden (CFU/ml CSF) (IQR)	11,200 (390–63,000)
Mortality [no. (%) of patients who died/total no. of patients]	
2 wk	1/16 (6)
10 wk	4/12 (33)^*c*^
1 yr	5/12 (42)

a IQR, interquartile range.

b GCS, Glasgow coma scale.

c Four patients were lost to follow-up at between 2 and 10 weeks.

A three-compartment pharmacokinetic model consisting of gut, plasma, and CSF was used to describe the concentration-time course of fluconazole administered orally or i.v. in the plasma and CSF (Table 3). Of the 17 patients, one was excluded because there was only a single PK measurement. The remaining 16 patients were well described by this model, with coefficients of determination (*r*^2^) for the linear regression of observed-predicted values after the Bayesian step for the plasma and CSF of 0.58 and 0.77, respectively. The Bayesian posterior estimates for each patient were used to calculate the percent partitioning into CSF. The mean partitioning of total drug fluconazole from the plasma into the CSF ± standard deviation was 94.54% ± 29.59%, with a range of 46.38 to 170.97%. With correction for protein binding, the partitioning was 106.22% ± 33.24% The shape of the concentration-time curve in the CSF was similar to that in plasma, without evidence of hysteresis (data not shown).

**TABLE 3:** PK parameters for 16 patients with >1 fluconazole PK data sample available from plasma and CSF

Parameter^*a*^	Value		
	Mean	Median	SD
*K_a_* (h^−1^)	4.383	1.031	6.483
SCL (liters/h)	0.794	0.624	0.303
*V* (liters)	18.136	10.464	11.302
Kcp (h^−1^)	10.341	0.260	12.674
Kpc (h^−1^)	15.603	14.915	7.905
Kcs (h^−1^)	27.805	27.189	13.131
Ksc (h^−1^)	35.756	39.671	10.776
*V*csf (liters)	16.293	13.425	14.369
*F*	0.903	1.000	0.152

a Parameters are as follows: *K_a_* is the first-order rate constant that connects the gut to the bloodstream, SCL is the first-order clearance of fluconazole from the central compartment (i.e., bloodstream), *V* is the volume of the central compartment, Kcp and Kpc are the first-order intercompartmental rate constants that connect the central and peripheral compartments, Ksc and Kcs are the first-order intercompartmental rate constants that connect the central and CSF compartments, *V*csf is the volume of the CSF, and *F* is the oral bioavailability of fluconazole.

A subset of 11 patients had concomitantly collected pharmacodynamic data following prespecified lumbar punctures (LPs) as well as additional procedures for management of raised intracranial pressure. Patients had a median of 3 lumbar punctures (range, 1 to 7) within the first 14 days of treatment, with limited numbers of observations for patients dying early. Details of the MICs and clinical outcomes are summarized in Table 4. The pharmacodynamic model fitted to these data is shown in Table 5. The MICs of fluconazole for clinical isolates from these lumbar punctures varied with the methodology used and timing of the test, i.e., immediate Etest performed on-site in Tanzania versus broth microdilution following several freeze-thaw cycles. Etest MICs were consistently higher than microdilution MICs, with modal MICs of 4 versus 1 mg/liter, respectively.

**TABLE 4:** Demographics, MICs, and clinical outcomes of the 11 patients in the pharmacokinetic-pharmacodynamic substudy

Patient	Patient in study by Stone et al.^*a*^	Age (yr)	Sex	MIC (mg/liter) (by broth microdilution)	Outcome^*b*^
A	001	19	F	2	Alive at 12 mo
B	002	35	M	1	Alive at 12 mo
F	007	37	M	1	Died at 3 mo
G	008	33	F	1	Alive at day 14 and then LFU
H	009	40	M	1	Died at 3 mo
J	010	48	F	1	Alive at day 11 and then LFU
K	011	42	M	1	Culture negative at day 14; died at 1 mo
N	014	31	F	1	Alive at day 5 and then LFU
O	015	50	F	0.25	Alive at 12 mo
Q	017	45	F	0.25	Alive at 2 wk and then LFU
R	018	40	F	2	Died at 1 mo

a See reference 13.

b LFU, lost to follow-up.

**TABLE 5:** Pharmacodynamic parameters for 11 patients with >1 estimate of fungal density from CSF

Parameter^*a*^	Value		
	Mean	Median	SD
popmax (CFU/ml)	9,066,008.322	9,986,903.386	2,745,844.217
kkmax_s (log_10_ CFU/ml/h)	0.101	0.035	0.114
C_50_k_s (mg/liter)	35.563	37.687	13.324
Hk_s	12.409	13.691	6.858
Kgmax_r (log_10_ CFU/ml/h)	0.031	0.030	0.001
kkmax_r (log_10_ CFU/ml/h)	0.039	0.041	0.013
C_50_k_r (mg/liter)	14.702	10.897	12.898
Hk_r	18.020	18.510	1.882
Initial Condition_s (CFU/ml)	89,543.363	11,218.526	133,792.013
Initial Condition_r (CFU/ml)	304.542	94.955	373.212

a Parameters are as follows: popmax is the maximum theoretical fungal density in CSF; kkmax_s is the maximum rate of fluconazole killing of the susceptible subpopulation in CSF; C_50_k_s is the CSF concentration of fluconazole at which the rate of killing of the susceptible subpopulation is half maximal; Hk_s is the slope function for the effect of fluconazole on the susceptible population; Kgmax_r is the maximum rate of growth of the resistant subpopulation; kkmax_r is the maximum rate of fluconazole killing of the resistant subpopulation in CSF; C_50_k_r is the CSF concentration of fluconazole at which the rate of killing of the resistant subpopulation is half maximal; Hk_r is the slope function for the effect of fluconazole on the resistant population; and Initial Condition_s and Initial Condition_r are the estimated initial densities of the susceptible and resistant subpopulations in the CSF, respectively.

**FIG 4:**
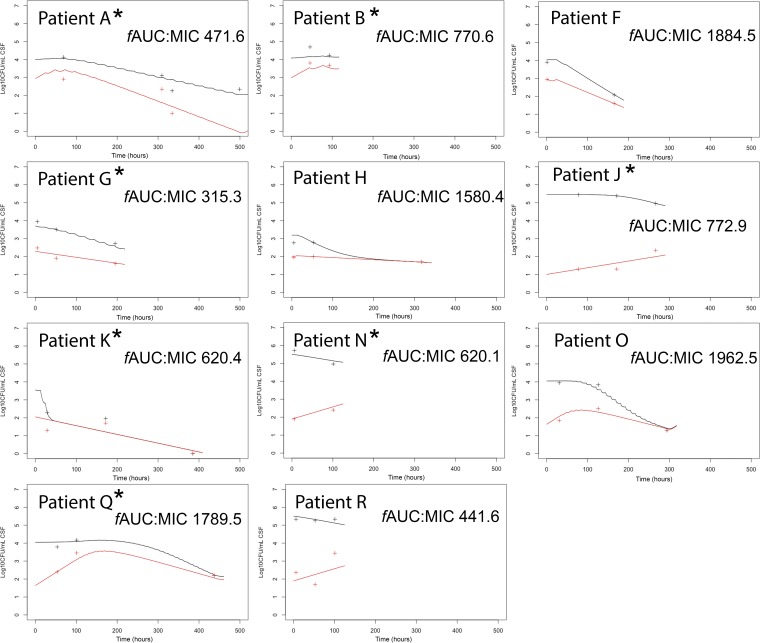
Time course of pharmacodynamics for patients with cryptococcal meningitis receiving fluconazole monotherapy at 800 to 1,200 mg/day i.v. and orally. The *f*AUC:MIC in CSF is shown for each patient. The black and red lines are Bayesian posterior predictions for the total and resistant populations (i.e., able to grow on agar containing 32 mg/liter fluconazole), respectively. Similarly, the black and red crosses are the observations from total and resistant populations from each patient, respectively. No patients died within the study period. Patients whose baseline, pretreatment isolates were found to have a resistant subpopulation growing on fluconazole-containing agar with aneuploidy after whole-genome sequencing are marked with an asterisk. The patients here (and those described previously by Stone et al. [13]) are designated patients A (patient 001 in the study by Stone et al.), B (002), F (007), G (008), H (009), J (010), K (011), N (014), O (015), Q (017), and R (18). Note that time zero is the time when the patient initially received fluconazole.

The raw pharmacodynamic data from each patient (patients A to R) are shown in Fig. 4 (note that these data have been previously reported [13]). The PK and PD data from the 11 patients were sufficient to fit a PK-PD model that described the time course of both the total (Fig. 4, black lines) and resistant (red lines) fungal subpopulations. Fluconazole did not achieve sterilization in 10/11 patients, with persistence of an underlying resistant subpopulation able to grow on agar containing fluconazole at 32 mg/liter. There was, however, considerable heterogeneity between patients. In the majority, the resistant subpopulation declined with fluconazole therapy, and in patients J, N, and R, there was an expansion of the resistant subpopulation, whereas in patients H, K, and O, the total population consisted entirely of a resistant subpopulation after the susceptible population was killed (Fig. 4). The *f*AUC:MIC in CSF for each patient was calculated using the MIC obtained using broth microdilution. Some of the variability in pharmacodynamics and expansion of a resistant clone(s) despite the attainment of an *f*AUC:MIC of >305 may be due to strain-to-strain differences in heteroresistance, mutational frequency, and the distribution of the MICs of emergent less susceptible subpopulations. Hence, there are clearly limitations in the use of a single MIC estimate that is assumed to be invariant for the pharmacodynamic analyses.

**FIG 5:**
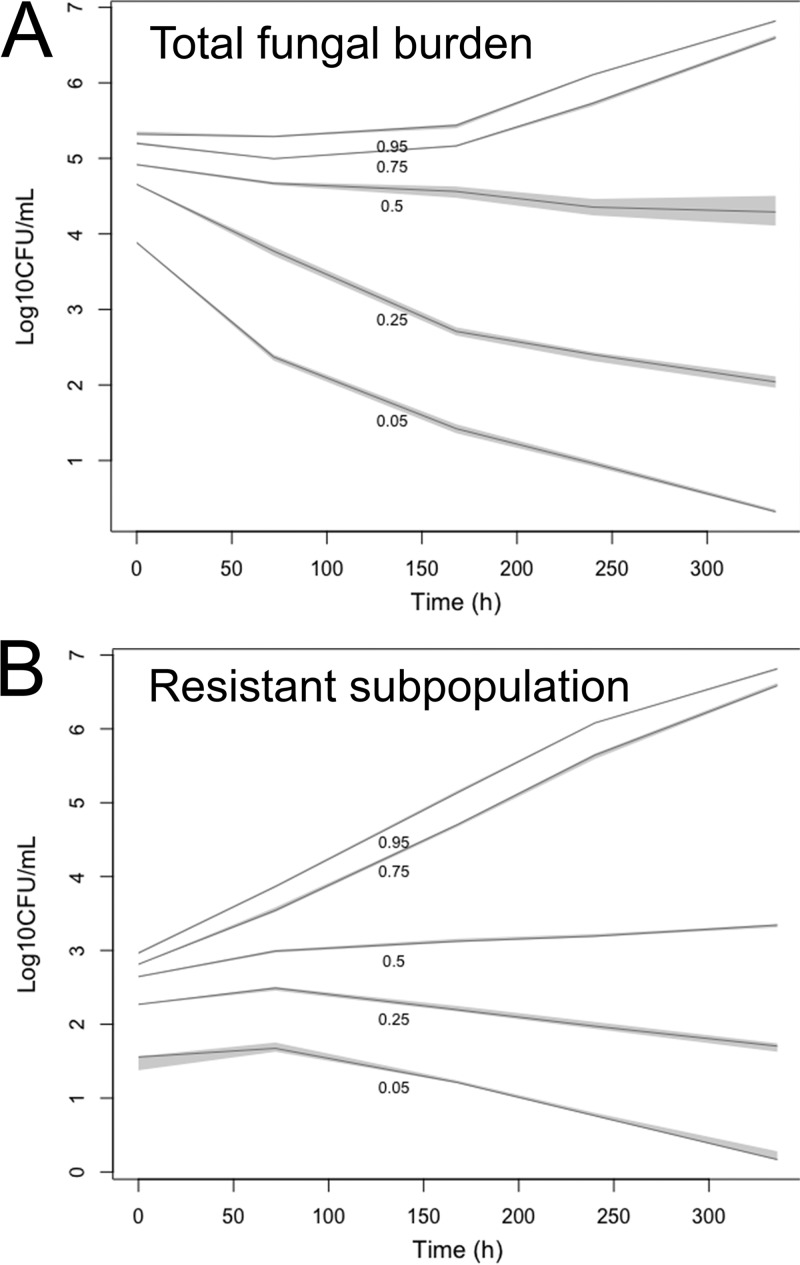
Summary of the Monte Carlo simulations constructed using the PK-PD data from the clinical study in Fig. 4. The 5th, 25, 50th, 75th, and 95th percentiles of the simulated population for the total fungal burden are shown (A), along with those of a resistant subpopulation growing on agar containing fluconazole at 32 mg/liter (B). The gray-shaded areas show the 95% confidence intervals around each percentile. A total of 12.8% of simulated patients are predicted to have sterilized CSF for total and resistant populations of C. neoformans. Resistant colonies were detectable (log_10_ CFU of >1) at various densities in 83.4% of patients.

Monte Carlo simulations performed in Pmetrics (20) allowed the potential consequences of a much larger population of patients receiving 1,200 mg fluconazole daily to be explored. More specifically, the simulations provided an estimate of the proportion of simulated patients that achieved sterilization after induction therapy and the variability of the fungal density-versus-time plots for both the total population and the resistant subpopulation. Simulations performed with the PK-PD model highlighted that the median fungal density-versus-time profile showed only a minor decline in fungal density with time (Fig. 5). Furthermore, only 12.8% of simulated patients achieved sterilization (i.e., log_10_ CFU of <1/ml CSF) at 14 days (336 h) posttherapy. Resistant colonies were detectable (log_10_ CFU of >1/ml CSF) at various densities in 83.4% of patients at the end of induction therapy. Hence, the simulations reflected the PK-PD data from the Tanzanian clinical study (9% sterilization) and were comparable with microbiological data from two prior clinical African cohorts receiving fluconazole monotherapy at 1,200 mg/day showing 2-week CSF sterilization in 6% and 18% of patients, respectively (3, 4).

## DISCUSSION

This pharmacokinetic-pharmacodynamic study demonstrates that fluconazole at clinically relevant exposures fails to sterilize the CNS and is ineffective at preventing the rapid emergence of resistance. Aneuploidy is the predominant mechanism, although other mechanisms are also likely to be operational. The study is unique in that it combines information from experimental models of cryptococcal meningitis with data from patients receiving fluconazole as monotherapy, which remains the norm in much of Africa despite not being consistent with current treatment recommendations from the WHO (1). The relatively poor microbiological clearance and ultimate clinical outcome following induction with fluconazole as a single agent have traditionally been attributed to a fungistatic pattern of killing. Here, we show that part of the reason for this microbiological failure is related to the emergence of resistance and that this in turn can be linked to the magnitude of fluconazole drug exposure.

The information on the pharmacodynamics of fluconazole obtained from the different model systems was complementary. The hollow-fiber infection model enabled the full range of pharmacodynamic responses to be delineated and suggests that an *f*AUC:MIC of ≥305.7 in CSF results in suppression of resistance. An inverted U suggests that it may be at least theoretically possible to generate fluconazole exposures in CSF that are sufficiently high to achieve fungicidal activity and minimize the emergence of resistance, although this would likely be at the expense of increased toxicity. A trial examining higher dosages of fluconazole (1,600 and 2,000 mg/day [ClinicalTrials.gov identifier NCT00885703]) has completed enrollment, and preliminary results are available (https://clinicaltrials.gov). If higher dosages were to be associated with improved microbiological and clinical outcomes, further PK-PD studies would be required to confirm the pharmacokinetic linearity of fluconazole, the linear partitioning of fluconazole into the CSF, and redefinition of the pharmacodynamics of fungal killing and the emergence of resistance.

Aneuploidy, particularly duplication of chromosome 1, is consistently observed in Cryptococcus neoformans following exposure to fluconazole. This mechanism is apparent in *in vitro* and murine models (12) as well as in patients with cryptococcal meningitis (13). Aneuploidy may result in fluconazole resistance via increased expression of the *ERG11* (drug target) and *AFR1* (efflux pump) genes, both of which are located on chromosome 1 (13). There are two possible scenarios that lead to the emergence of resistance and dominance of a clone with aneuploidy. First, there may be an expansion of a subpopulation present prior to fluconazole therapy (i.e., heteroresistance). The expansion is enabled by the killing of a fluconazole-susceptible subpopulation and growth (or persistence) of a resistant subpopulation. This pattern was exhibited in the hollow-fiber experiments where the resistant subpopulation present prior to fluconazole treatment progressively expanded and ultimately formed the majority of the fungal population. This is a function of whether the fungal density at treatment initiation is greater than the mutational frequency of resistance. In mice, for instance, the estimated burden in brain at the time of treatment initiation was approximately 100 CFU/g cerebrum (Table 1), which is well below the mutational frequency. This may explain why resistance did not emerge in all murine experiments but could potentially be circumvented by inoculation directly into the CNS. In the second scenario, there may be a development of *de novo* resistance (albeit with the same molecular mechanism). The second scenario may occur without the presence of a resistant subpopulation prior to drug exposure.

Aneuploidy formation is a general Hsp90 stress-induced phenomenon in eukaryotes, including fungi, capable of fueling rapid phenotypic evolution, for which drug resistance is just one phenotype (21). The emergence of chromosome 1 aneuploidy that was also seen in vehicle-treated controls supports this hypothesis. Fluconazole has been shown to promote aneuploidy in both Candida albicans and C. neoformans, albeit via different mechanisms, resulting in reduced antifungal susceptibility (22–24). Fluconazole treatment induced the formation of chromosome 1 disomy by the end of the experiment using the H99 reference strain in both the HFIM and murine models, and chromosome 1 disomy was the most commonly emergent aneuploidy in the fluconazole-treated clinical cohort (13). There are some challenges in definitively linking the emergence of resistance (as defined by growth on drug-containing media) with aneuploidy. Selecting colonies from drug-free media risks missing a resistant subpopulation with aneuploidy. Conversely, selecting colonies from drug-containing media may lead to erroneous conclusions if the presence of fluconazole induces aneuploidy, but the primary reason for resistance was an alternative mechanism.

The plasticity and instability of both resistance genotype and phenotype have hampered attempts to understand the links between fluconazole drug exposure, microbiological failure, and the emergence of antifungal resistance. Such plasticity is difficult to model using current PK-PD paradigms where the MIC is assumed to be invariant. Previous studies have largely failed to demonstrate a relationship between fluconazole MIC for C. neoformans and clinical outcomes (14, 25–27). Retesting of clinical isolates from patients failing fluconazole therapy with high MICs generally results in MICs in the wild-type range. Moreover, there are likely to be differences in MICs according to whether microdilution or the Etest is used. We found that isolates retested using broth microdilution following frozen shipment and a single passage had on average 2- to 4-fold-lower MICs than those originally tested in Tanzania using the Etest. These discrepancies pose a challenge to the construction of PK-PD relationships and PK-PD bridging studies with conclusions dependent on MIC methodology and the way in which the isolate is prepared prior to testing.

This study has several limitations. Only a single challenge strain was used for the HFIM and murine studies. The clinical data set was relatively small, with fewer patients still with a full complement of PK-PD data. Resistance was defined as a dichotomous event (ability to grow on medium containing 32 mg/liter fluconazole) when, in reality, there is a population of patients infected with cryptococcal isolates with a range of MICs. While chromosome 1 disomy in H99 consistently emerged in all contexts, additional aneuploidies were also observed. Furthermore, as-yet-undefined mechanisms other than aneuploidy may be present (13). The clinical PK-PD analysis was exclusively based on fluconazole concentrations in CSF, which is only one subcompartment in the central nervous system. The clinical pharmacodynamics of fluconazole for fungal infection within the cerebrum remain difficult to define but may be reasonably different from those in CSF and ultimately may have a significant impact on clinical outcomes (28).

Despite fluconazole being a safe, widely available, and highly orally bioavailable antifungal agent with extensive partitioning into CSF, the antifungal activity of fluconazole for cryptococcal meningitis is compromised by the emergence of resistance. This is a dynamic, intrinsic property that may be poorly predicted by standard MIC testing at the initiation of treatment. Dosage escalation may be required to achieve sufficient exposure to suppress the resistant subpopulation, but the clinical efficacy and tolerability of such a strategy require further study. Combination chemotherapy is an additional way in which the emergence of resistance can be addressed, but clinical choices are currently limited to 5FC. The applicability of our findings to other triazoles, such as posaconazole, voriconazole, and isavuconazole, or other compounds that utilize 14-α-demethylase as their target (e.g., VT1598) is unknown. Our findings underscore the urgent need for the development of new agents and combinations to reduce the global toll of cryptococcal meningitis.

## MATERIALS AND METHODS

### Strains of C. neoformans and *in vitro* susceptibility to fluconazole.

H99 (ATCC 208821) was used as the challenge strain for the hollow-fiber infection models and the murine model of meningoencephalitis. The MICs of fluconazole against H99 (ATCC 208821) and the clinical isolates were estimated using broth microdilution methodology of the Clinical and Laboratory Standards Institute (17).

### Hollow-fiber infection model.

A recently described hollow-fiber infection model (HFIM) of cryptococcal meningitis was used. Briefly, hollow-fiber cartridges (FiberCell Systems, Frederick, MD, USA) were used and configured as previously described (16). The extracapillary space of each cartridge was inoculated with 40 ml of a suspension containing approximately 6 log_10_ CFU/ml of C. neoformans var. *grubii* (ATCC 208821; H99). Yeast extract-peptone-dextrose (YPD) medium was pumped from the central compartment through the cartridge and back again using a peristaltic pump (205 U; Watson-Marlow, United Kingdom). The HFIM was incubated at 37°C in ambient air. Human-like concentration-time profiles in CSF were simulated and designed to encapsulate the pharmacodynamics of antifungal activity and the emergence of resistance. The time course of fungal growth was determined by removing 1 ml from the extracapillary space of the cartridge and plating serial 10-fold dilutions onto both drug-free YPD agar and YPD agar containing 32 and 64 mg/liter fluconazole.

### Murine model of meningoencephalitis.

A well-characterized murine model of disseminated infection was used. Male CD1 mice weighing approximately 25 g at the time of the experiment were injected i.v. with 0.2 ml of a phosphate-buffered saline (PBS) suspension containing 3 × 10^8^ CFU/ml (i.e., inoculum of 6 × 10^7^ CFU per mouse). No immunosuppression was used because mice are intrinsically susceptible to invasive infection caused by C. neoformans.

The *in vivo* pharmacokinetic (PK) studies were performed on infected mice using 125 and 250 mg/kg/day. Treatment with fluconazole commenced at 24 h postinoculation. Fluconazole was administered daily by oral gavage. A serial-sacrifice design was used to define the PK. Blood was collected by cardiac puncture under anesthesia with 1% isoflurane, followed by CO_2_ asphyxia. Plasma was obtained by centrifugation and stored at −80°C until analysis. Plasma samples were obtained at 0.5, 1, 2, 4, 6, and 24 h postdose in the first (i.e., 24 to 48 h postinoculation) and third (i.e., 72 to 96 h postinoculation) dosing intervals. Following sacrifice, the cerebrum was removed, homogenized in PBS, and stored at −80°C until analysis.

For the *in vivo* murine pharmacodynamic studies, groups of 3 mice were sacrificed immediately postinoculation and then at 24, 48, 96, 144, 192, and 240 h postinoculation. The cerebrum was removed and plated onto drug-free agar and agar containing fluconazole at 32 mg/liter.

### Clinical study.

A clinical PK-PD study was performed at the Muhimbili National Hospital in Dar es Salaam, Tanzania, as a run-in study to the ACTA trial, which was recently reported (29). Ethical approval was obtained from the National Institute of Medical Research (NIMR) in Tanzania as well as the London School of Hygiene and Tropical Medicine Ethics Committee (reference number 9176). Informed consent was obtained from all patients or their guardians. Patients were eligible for enrollment if they were >18 years of age with an initial episode of cryptococcal meningitis, confirmed positive for cryptococcal antigen (CrAg) in serum or CSF (Immy, Norman, OK), a positive cryptococcal culture, and/or positive India ink stain in CSF. Patients who were pregnant, unable to receive fluconazole for any reason, or already on fluconazole therapy for >48 h were excluded. Patients received fluconazole as induction therapy at 800 to 1,200 mg in one or two divided dosages, according to local treatment policies. This was ethically permissible because in Tanzania, as in much of Africa, the gold-standard induction treatment of amphotericin B deoxycholate and flucytosine is not available. Induction therapy with fluconazole is the standard of care for cryptococcal meningitis and all other clinical forms of cryptococcosis.

Consent was sought for plasma sampling at multiple time points. Because permission for intensive preplanned sampling was often refused, the majority of plasma samples were acquired opportunistically when samples were obtained as part of routine care. Patients consented to lumbar punctures (LPs) at baseline and at days 7 and 14 of treatment. Additional LPs were performed if clinically indicated for the management of raised intracranial pressure. These samples were processed for PK and microbiological analysis. For every CSF sample, the magnitudes (log_10_ CFU per milliliter of CSF) of the total fungal density and the density of the resistant subpopulation were quantified by directly plating CSF onto drug-free and fluconazole-containing agar plates at a final concentration of 32 mg/liter as previously described (13). Samples of plasma and CSF for measurement of fluconazole were prepared by centrifugation. The resultant supernatant was stored at −80°C before being shipped to the University of Liverpool for measurement of fluconazole concentrations. C. neoformans MIC determinations were performed on-site using the fluconazole Etest (bioMérieux, Boston, MA) and subsequently using broth microdilution as described above.

### Measurement of fluconazole in experimental and clinical samples.

Fluconazole concentrations were measured using a validated liquid chromatography-tandem mass spectrometry (LC-MS/MS) method with a 1260 Agilent ultraperformance liquid chromatography (UPLC) system coupled to an Agilent 6420 Triple Quad mass spectrometer (Agilent Technologies UK Ltd., Cheshire, UK). Briefly, fluconazole was extracted by protein precipitation: 300 μl of cold methanol containing the internal standard fluconazole-D4 at 0.625 mg/liter (TRC, Canada) was added to 10 μl of the sample (plasma or CSF). The solution was vortex mixed for 5 s and filtered through a Sirocco precipitation plate (Waters Ltd., Cheshire, UK). One hundred fifty microliters of the supernatant was transferred to a 96-well autosampler plate, and 3 μl was injected into an Agilent Zorbax C_18_ rapid resolution high definition (RRHD) (2.1 by 50 mm; 1.8 μm) (Agilent Technologies UK Ltd., Cheshire, UK).

Chromatographic separation was achieved using a gradient consisting of 70% mobile phase A–30% mobile phase B (0.1% formic acid in water as mobile phase A and 0.1% formic acid in methanol as mobile phase B). The organic phase was increased to 100% over 90 s, with an additional 90 s of equilibration.

The mass spectrometer was operated in a multiple-reaction-monitoring scan mode in positive polarity. The precursor ions were *m/z* 307.11 and *m/z* 311.1 for fluconazole and the internal standard, respectively. The product ions for fluconazole were *m/z* 220.1 and *m/z* 238.1, and those for the internal standard were *m/z* 223.2 and *m/z* 242.1. The source parameters were set as follows: capillary voltage of 4,000 V, gas temperature of 300°C, and nebulizer gas at 15 lb/in^2^.

The standard curve for fluconazole encompassed the concentration range of 1 to 120 mg/liter and was constructed using a blank matrix. The limit of quantitation was 1 mg/liter. In plasma, the intraday coefficient of variation (CV) was <3.4%, and the interday CV was <6.7%, over the concentration range of 1 to 90 mg/liter. In CSF, the intraday CV was <5.2%, and the interday CV was <5.3% over the same concentration range.

### Sequencing and bioinformatics.

Genomic DNA was extracted using the MasterPure yeast DNA purification kit (Epicentre, Madison, WI). Following resuspension in lysis buffer and the addition of RNase, two rounds of cell disruption were performed using a FastPrep homogenizer and lysing matrix C (MP Biomedicals, Solon, OH). The resulting lysate was centrifuged at 12,000 × *g* for 2 min, and the supernatant was heated at 65°C for 15 min. Protein precipitation and DNA recovery were performed according to the manufacturer’s instructions. A further round of RNase treatment was performed, and DNA was purified using the genomic DNA clean and concentrator kit (Zymo Research, Irvine, CA).

Libraries were constructed from submitted samples using the Illumina TruSeq Nano DNA HT library prep kit. Briefly, 100 ng DNA for each sample was sheared to an average of 350 bp using the Bioruptor Pico sonicator (Diagenode) and cleaned, and the products were A tailed by incubation at 37°C for 30 min and ligated to dual-ended adapters at 30°C for 10 min. Template DNA was diluted to 3 nM, 5 μl was denatured for 8 min at room temperature using 5 μl freshly diluted 0.1 N sodium hydroxide (NaOH), and the reaction was subsequently terminated by the addition of 5 μl 0.1 M Tris Cl (pH 8). The final loading concentration of 300 pM was reached by adding 35 μl exclusion amplification enzyme mix. Clustering of DNA templates was performed using a HiSeq 3000/4000 paired-end (PE) cluster kit with a cBot cluster generation system (Illumina), according to the manufacturer’s instructions. Following cluster generation, libraries were sequenced on an Illumina HiSeq 4000 instrument (Illumina) with version 1 chemistry using sequencing-by-synthesis (SBS) technology to generate 2- by 150-bp paired-end reads.

A total of 34 samples (*n* = 15 for the murine study and *n* = 19 for the HFIM) were sequenced on the Illumina HiSeq4000 platform (2- by 150-bp paired-end reads) (Illumina, Inc., San Diego, CA) by the Centre for Genomic Research (https://www.liverpool.ac.uk/genomic-research), University of Liverpool, UK. Base calling and demultiplexing of indexed reads were performed by using CASAVA version 1.8.2 (Illumina, Inc., San Diego, CA) to produce the raw sequence data in FASTQ format. The raw FASTQ reads were trimmed to remove Illumina adapter sequences using Cutadapt version 1.2.1 (30) and low-quality bases using Sickle version 1.200 (31).

Trimmed reads were aligned to the Cryptococcus neoformans var. *grubii* H99 reference genome (https://fungi.ensembl.org/Cryptococcus_neoformans_var_grubii_h99/Info/Index) with the short-read alignment tool BWA-MEM (version 0.7.5a-r405) (32). Following alignment, PCR and optical duplicate reads were identified and removed with Picard (version 1.94) (http://broadinstitute.github.io/picard). Subsequently, the Genome Analysis Toolkit (GATK) (version 3.7) (33) Indel Re-aligner module (34) was used to locally realign reads around putative insertion and deletion sites. The aligned data were then analyzed using Control-FREEC (35) to assess chromosomal ploidy.

### PK-PD modeling.

The clinical PK-PD study was modeled in a 2-step process to try to provide a stable solution from relatively sparse data. First, the PK was solved using the following sets of inhomogeneous differential equations that described the transfer of fluconazole from the gut (compartment 1) to the central bloodstream (compartment 2) and the peripheral compartment (compartment 3) and into the CSF (compartment 4). Fluconazole was administered either as a bolus [B(1)], as a tablet, or as an i.v. infusion [R(1)], which enabled bioavailability (*F*) to be estimated (not shown in the differential equations).

$$\text{XP(1)}=\text{B(1)}-K_{a}\times \text{X(1)}$$

$$\text{XP(2)}=\text{R(1)}+K_{a}\times \text{X(1)}-\text{SCL}/V\times \text{X(2)}-\text{Kcp}\times \text{X(2)}+\text{Kcp}\times \text{X(3)}-\text{Kcs}\times \text{X(2)}+\text{Ksc}\times \text{X(4)}$$

XP(3)=Kcp×X(2)−Kcp×X(3)

XP(4)=Kcs×X(2)−Ksc×X(4)

The model outputs are denoted by Y(1) and Y(2), and the two output equations are denoted by

Y(1)=X(2)/V

Y(2)=X(4)/Vcsf

with the first and second output equations representing the time course of plasma and CSF concentrations of fluconazole, respectively. The amount of drug in compartment 1, 2, 3, and 4 is denoted by X(1), X(2), X(3), and X(4), respectively. Similarly, the rate of change of amount in each compartment is denoted by XP(1), XP(2), XP(3), and XP(4), respectively. The *f*AUC in plasma and CSF was calculated using integration from the Bayesian posterior parameter values.

The median Bayesian posterior estimates described each individual patient well. These were obtained and fixed for the pharmacodynamic modeling below. For pharmacodynamic modeling, the following model was used. The first 4 equations are the same as the ones described above and describe the PK in the gut, plasma, peripheral compartment, and CSF. The time courses of the densities of the susceptible and resistant subpopulations are described by the fifth and sixth equations, respectively. The structure of these equations differed slightly. The growth of a susceptible population was not observed (since it is unethical to withhold therapy). Hence, the density of this subpopulation could only decrease with time from an initial starting value, which was estimated in the fitting process as an initial condition. In contrast, both the growth and death of the resistant subpopulation were observed. Hence, the equation contains a term that describes the capacity for limited growth as well as fluconazole-induced killing that is explicitly linked to concentrations within the CSF. An initial density of the resistant population was estimated in the same way as for the susceptible subpopulation.

$$\text{XP(5)}=-\text{kkmax\_s}\times [\text{X(4)}/V\text{cns}]**\text{Hks}/\{{\text{C}}_{50}\text{k\_s}**\text{Hk\_s}+[\text{X(4)}/V\text{cns}]**\text{Hk\_s}\}\times \text{X(5)}$$

$$\text{XP(6)}=\text{Kgmax\_r}\times \{1\text{.D}0-[\text{X(6)}/\text{popmax}]\}\times \text{X(6)}-\text{kkmax\_r}\times [\text{X(4)}/V\text{cns}]**\text{Hk\_r}/\{{\text{C}}_{50}\text{k\_r}**\text{Hk\_r}+[\text{X(4)}/V\text{cns}]**\text{Hk\_r}\}\times \text{X(6)}$$

The two output equations for the pharmacodynamics were

Y(1)=DLOG10[X(5)+X(6)]

Y(2)=DLOG10[X(6)]

The first equation describes the time course of the total population (i.e., susceptible plus resistant). The second equation describes the time course of the resistant subpopulation. DLOG10 is the logarithm base 10 of the fungal density.

### Monte Carlo simulations.

Simulations were performed using the linked PK-PD model. Because the original problem was solved in a two-step process, the PK model was used to generate 100 simulated patients, each with their own unique set of PK parameters. These simulated patients were then supplied to the pharmacodynamics portion of the mathematical model, and each set of PK parameters was then used to generate 100 further simulated patients with pharmacodynamic outputs. Data from these simulated patients were used to define the time courses of fungal densities of the total and resistant subpopulations in the CSF for the 5th, 25th, 50th, 75th, and 95th percentiles of the simulated population.
