# Supplementary figures and images for: Fluconazole Monotherapy Is a Suboptimal Option for Initial Treatment of Cryptococcal Meningitis Because of Emergence of Resistance
Source: mBio. 2019 Dec 3;10(6):e02575-19. doi: 10.1128/mBio.02575-19 (PMC6890991; doi:10.1128/mBio.02575-19)

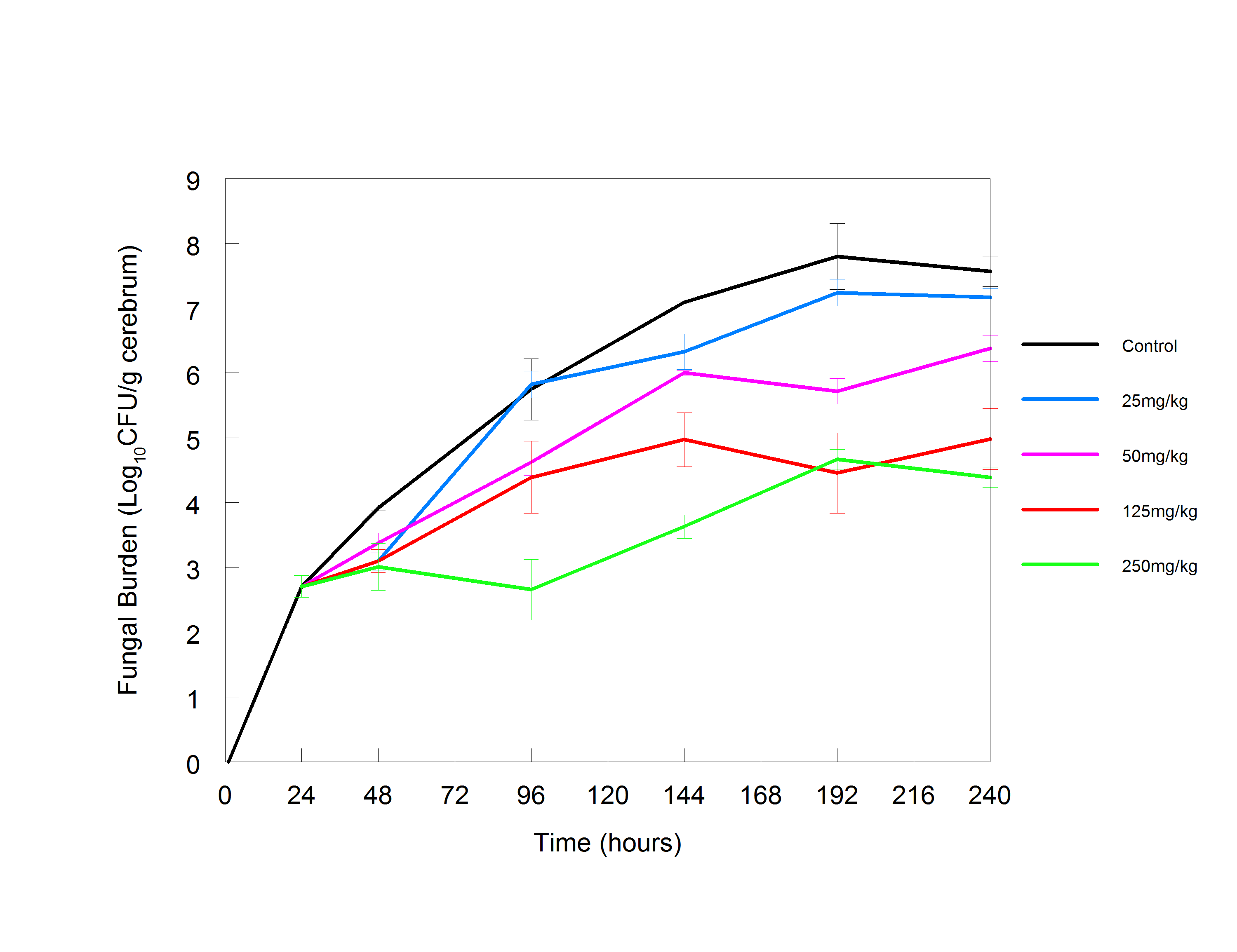

Supplement: FIG S1 [file mBio.02575-19-sf001.tif]

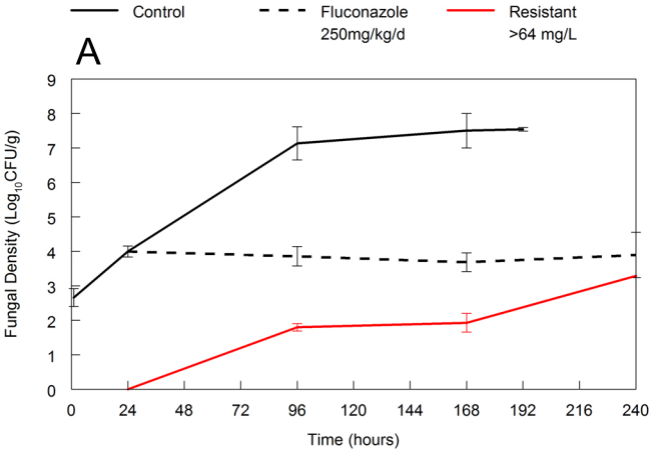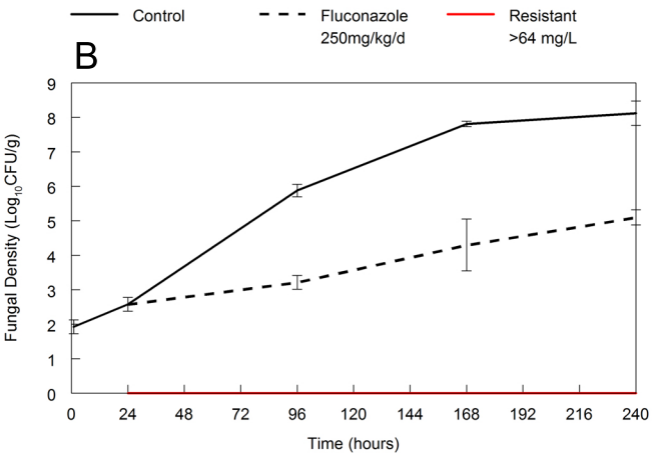

Supplement: FIG S2 [file mBio.02575-19-sf002.pdf]
